# Recommendations for fluid management of adults with sepsis in sub-Saharan Africa: a systematic review of guidelines

**DOI:** 10.1186/s13054-020-02978-4

**Published:** 2020-06-05

**Authors:** Benjamin Silberberg, Stephen Aston, Selda Boztepe, Shevin Jacob, Jamie Rylance

**Affiliations:** 1 Aintree University Hospital, Liverpool University Hospitals NHS Foundation Trust, Liverpool, L9 7AL UK; 2 Department of Biostatistics, University of Liverpool, Liverpool, UK; 3 Institute of Infection and Global Health, University of Liverpool, Liverpool, UK; 4 University Hospitals Bristol NHS Foundation Trust, Bristol, UK; 5 Department of Clinical Sciences, Liverpool School of Tropical Medicine, Liverpool, UK

**Keywords:** Sepsis, Fluid therapy, Vasoconstrictor agents, Africa south of the Sahara, Practice guidelines as topic, Systematic review

## Abstract

**Background:**

Sepsis guidelines are widely used in high-income countries and intravenous fluids are an important supportive treatment modality. However, fluids have been harmful in intervention trials in low-income countries, most notably in sub-Saharan Africa. We assessed the relevance, quality and applicability of available guidelines for the fluid management of adult patients with sepsis in this region.

**Methods:**

We identified sepsis guidelines by systematic review with broad search terms, duplicate screening and data extraction. We included peer-reviewed publications with explicit relevance to sepsis and fluid therapy. We excluded those designed exclusively for specific aetiologies of sepsis, for limited geographic locations, or for non-adult populations. We used the AGREE II tool to assess the quality of guideline development, performed a narrative synthesis and used theoretical case scenarios to assess practical applicability to everyday clinical practice in resource-constrained settings.

**Results:**

Published sepsis guidelines are heterogeneous in sepsis definition and in quality: 8/10 guidelines had significant deficits in applicability, particularly with reference to resource considerations in low-income settings. Indications for intravenous fluid were hypotension (8/10), clinical markers of hypoperfusion (6/10) and lactataemia (3/10). Crystalloids were overwhelmingly recommended (9/10). Suggested volumes varied; 5/10 explicitly recommended “fluid challenges” with reassessment, totalling between 1 L and 4 L during initial resuscitation. Fluid balance, including later de-escalation of therapy, was not specifically described in any. Norepinephrine was the preferred initial vasopressor (5/10), specifically targeted to MAP > 65 mmHg (3/10), with higher values suggested in pre-existing hypertension (1/10). Recommendations for guidelines were almost universally derived from evidence in high-income countries. None of the guidelines suggested any refinement for patients with malnutrition.

**Conclusions:**

Widely used international guidelines contain disparate recommendations on intravenous fluid use, lack specificity and are largely unattainable in low-income countries given available resources. A relative lack of high-quality evidence from sub-Saharan Africa increases reliance on recommendations which may not be relevant or implementable.

## Background

Sepsis is *life-threatening organ dysfunction due to a dysregulated host response to infection* [1]. Sepsis is common (48.9 million cases/year globally) and results in an estimated 11 million deaths annually [2]. In sub-Saharan Africa (SSA), where endemic tropical infections and advanced HIV are prevalent, models suggest the incidence of sepsis is higher (1527/100,000 cases per year compared with 678/100,000 globally) and represents 30–65% of overall mortality in the region, but primary sepsis-specific data are limited [3]. A recent systematic review of limited data from 15 studies (2800 participants from sub-Saharan Africa) estimated pooled in-hospital mortality for sepsis and severe sepsis at 19% (95% CI 12–29%) and 39% (95% CI 30–47%), respectively [4]. Strategies for improving sepsis survival in low-income countries (LIC) have been limited by lack of robust evidence, insufficient resources in emergency care and conflicting data from high- and low-income settings.

High-income countries have widely adopted guidelines developed by the Surviving Sepsis Campaign (SSC). Adherence to these guidelines in observational studies is associated with improved survival [5–7]. Recent meta-analysis of three large multicentre studies of early goal-directed therapy confirmed each study’s individual findings: whilst protocolised care increased use of intravenous fluids, vasoactive agents and blood products, it resulted in greater need for intensive care and renal replacement therapy and did not improve survival [8]. Where “bundles of care” cannot be entirely implemented (for example where centres in LIC typically have minimal access to mechanical ventilation or central venous access), there is considerable uncertainty about the value of component interventions, which frequently lack their own evidence base. For example, fluid resuscitation received only B and C level grades on GRADE criteria [9]. Furthermore, randomised controlled trials from Africa in adults and children have demonstrated the potential for harm using bolus fluids in LIC [10–12].

There are few specific data on the availability of medication, equipment and skilled personnel required to provide gold standard care to critically unwell patients in SSA. A survey of anaesthesia providers reported significant resource limitation in SSA compared to high-income countries (HIC) and considerable heterogeneity: over 25% of respondents’ hospitals had no intensive care facilities, fewer than one quarter could measure serum lactate and central venous pressure monitoring was possible in just over one third. Overall, 1.4% of hospitals in SSA had the necessary resources to implement the SSC guidelines in their entirety, compared with 81% of hospitals in HIC [13].

Guidelines for treatment of acute infection, including those for intravenous fluid administration, are widely used and referenced. Given the uncertainty over the safety and efficacy in Africa particularly, we aimed to systematically review the availability of published clinical guidelines which made recommendations on fluid use, to describe the source and target audience of the core recommendations and their practical applicability in typical LIC situations.

## Methods

We searched the Medline, PubMed and Web of Science databases in June 2017 using the search terms in Table 1. We included guidelines if they included recommendations for clinical management, with an explicit statement of applicability in sepsis or infection and were published after 1990 by one of the following: a peer-reviewed journal, international professional body or society. For revisions, the most recent iteration was included. Exclusion criteria were explicitly defining a scope which excludes sub-Saharan African populations, primarily pertaining to clinical practice on intensive care units or paediatric practice only (age < 16 years) or exclusively to specific aetiologies of sepsis (e.g. intra-abdominal sepsis), meta-analysis, case report and case series and no English translation available.

**Table 1:** Search terms

(“Sepsis”[Mesh] OR “Infection”[Mesh] OR sepsis[Title/Abstract] OR septic[Title/Abstract]) AND (“Infusions, Intraosseous”[Mesh] OR “Infusions, Parenteral”[Mesh] OR “Infusions, Intravenous”[Mesh] OR fluid*[Title/Abstract] OR intravenous[Title/Abstract] OR shock[Title/Abstract]) AND (“Practice Guideline” [Publication Type] OR “Guidelines as Topic”[Mesh] OR “Guideline” [Publication Type] OR guideline*[Title/Abstract] OR recommendation*[Title/Abstract]).	
We also sought expert opinion to identify existing guidelines and recommendations and review the reference list of relevant sources using a network of experts in the region and a cascading approach, including contacting the relevant national health ministries.	

Searches were screened for relevance by title and abstract and selected articles underwent full manuscript review. From eligible articles, data on sepsis definition, indications for initiation of fluid management, type and volume of fluid, assessment of response to fluid administration, criteria for cessation of fluid therapy, indications for initiation of vasopressor therapy and choice of initial vasopressor were extracted onto a proforma. Two reviewers (JR and BS) performed each stage in parallel and disagreement was resolved by consensus. Quality of the recommendations was assessed by the AGREE II tool [14] by the two independent reviewers. Where significant discrepancy existed between the reviewers’ scores, this was resolved by mutual consensus; the factors taken into consideration at this stage are described in the supplementary material. The AGREE II tool assigns a numerical score for the quality of guideline production and reporting within 6 domains: overall scope, stakeholder involvement, methodological rigour of the evidence analysis and synthesis, clarity of presentation, real-world relevance and applicability, and potential for editorial bias or competing interests.

The utility of practically implementing guidelines was assessed by applying the recommendations to predefined clinical scenarios. Three scenarios were designed which reflected aspects of treatment decisions faced by clinicians: (A) suspected infection with evidence of poor peripheral perfusion and altered mental status (severe sepsis according to Sepsis-2), when pertaining to a normal weight adult and a wasted adult; (B) suspected infection with hypotension and hypoxaemia; (C) suspected infection with raised lactate and comorbidities suggesting likely fluid intolerance (congestive cardiac failure). Guidelines were assessed by two doctors and disagreement resolved by consensus.

## Results

**Fig. 1:**
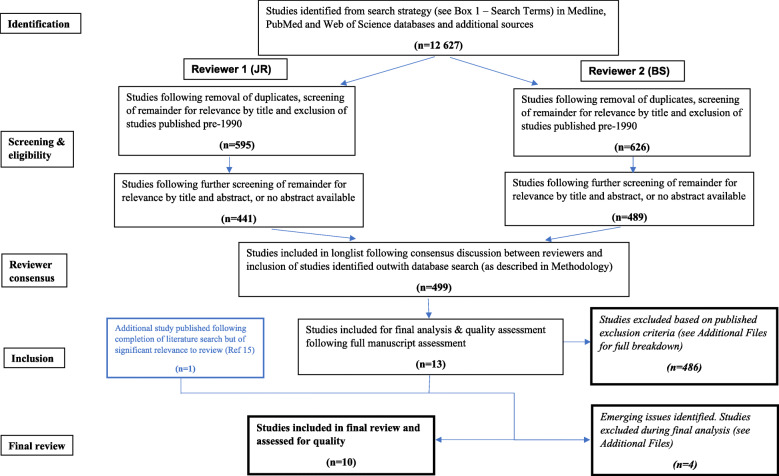
Literature search flowchart

An initial literature search, using the broad search terms defined in Table 1, identified over 12,000 studies. These were screened for full manuscript review (*n* = 499), of which 486 were excluded (Fig. 1). An additional study was identified from secondary searches [15]. Of 14 studies included for final analysis, 4 were excluded due to being not directly relevant (2), lacking full English translation (1) and including only secondary data (1)—see details in Additional file 1. Results from ten finally selected guidelines, published between 2004 and 2017, are summarised below.

### Quality assessment

A summary of quality assessment scores is given in Table 2, with full details of consensus scoring in the additional files. Two out of ten guidelines exceeded a score of 70% indicating highly rigorous and robust guideline development processes (NICE and Surviving Sepsis Campaign recommendations) which reflects the resources available to develop them [9, 16]. For each of the others, significant deficiency was noted in at least 2 domains, of which the most frequent concern (in 8 out of 10 of the guidelines) was the “Applicability” domain (whether consideration had been given to “the likely barriers and facilitators to implementation, strategies to improve uptake and resource implications of applying the guideline”) [14].

**Table 2:**
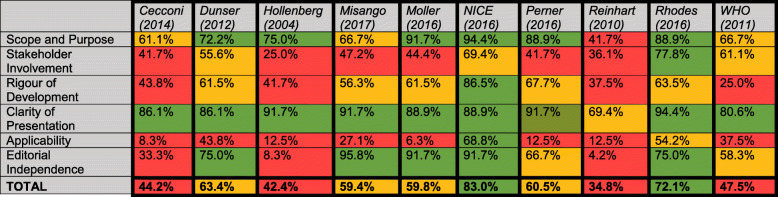
Combined AGREE II scores, by domain

Domain and combined scores are colour coded according to numerical score following AGREE-II assessment of guideline quality. Red indicates a score of >50% or poor performance; amber indicates a score of 50-70% or adequate performance; green indicates a score of >70% or good performance

### Sepsis definitions

The definitions used for sepsis varied (see Table 3) and were not explicit in three guidelines [15, 17, 18]. One provided a definition which was abstract and not directly clinically applicable [19]. Three defined bespoke criteria for sepsis [16, 20, 21] and three employed international definitions with and without modification [9, 22, 23].

**Table 3:** Sepsis definitions used by guidelines

Guideline	Definition
**Cecconi et al.** [19]	**“Life-threatening, generalized form of acute circulatory failure associated with inadequate oxygen utilization by the cells”**
**Dunser et al.** [22]	Sepsis-2^*^ modified **to replace criteria based on white blood cell count with “malaise and/or apathy”**
**Hollenberg et al.** [20]	**Haemodynamic support considered for hypoperfusion*(defined as systolic BP < 90 mmHg, MAP < 65 mmHg, fall of systolic BP > 40 mmHg, change in mental status, decrease in urine output, increased lactate)***
**NICE** [16]	**Clinical suspicion of infection, with risk criteria for death*(*e.g.*altered mental status, evidence of microvascular perfusion defect—mottled/delayed capillary refill time, high respiratory rate)***
**Reinhart et al.** [23]	Sepsis-2^*^
**Rhodes et al.** [9]	**Sepsis-3**^**†**^
**WHO** [21]	**Severe sepsis/septic shock defined as suspected infection plus hypotension (systolic BP < 90 mmHg) plus ≥ 1 of*pulse > 100 bpm, respiratory rate > 24, temperature < 36 °C or > 38 °C***
**Published definitions**	
*Sepsis-2 [24]	**Sepsis:** Proven or highly suspected infection plus presence of ≥ 2 of the following conditions: heart rate > 90 bpm, respiratory rate ≥ 20/min or PaCO2 < 32 mmHg, temperature < 36 °C or > 38 °C, white blood cell count < 4 × 10^6^ or > 12 × 10^6^ g/L or > 10% immature forms **Severe sepsis:** Sepsis *plus* confusion, hypoxaemia or elevated lactate
**†Sepsis-3** [1]	**Sepsis:** Life-threatening organ dysfunction caused by a dysregulated host response to infection **Organ dysfunction:** ≥ 2 points on qSOFA score, with 1 point scored for each of the following: respiratory rate > 22/min, altered mentation, systolic BP ≤ 100 mmHg

### Recommendations

We identified recommendations in the guidelines relating to indications for (1) initiating fluid management, (2) choice of type and volume of fluid, (3) criteria for assessing response to fluid administration, (4) criteria for cessation and (5) indications and choice for initial vasopressor treatment.

### Indications for intravenous fluid treatment

Two papers gave no specific recommendations on indications for intravenous fluid treatment, being practice guidelines for initial vasopressor therapy and choice of fluid, respectively [17, 18]. Of the remaining eight guidelines, hypotension was a common indication, with specific criteria in terms of systolic blood pressure (BP) described in four [9, 16, 20, 25]. A systolic BP of 90 mmHg was given as the threshold in all other than the Surviving Sepsis guidelines, where 100 mmHg was used as per the qSOFA scoring system. Suspected hypovolemia was referenced at an unspecified threshold in a further two guidelines [15, 23]. One guideline recommended treatment for patients with shock and explicitly stated that hypotension was not required to make this diagnosis, which should be based on a constellation of clinical findings (not specifically described) and lactate (> 2 mmol/l) [19].

Clinical manifestations of hypoperfusion, representing indications for commencement of fluid therapy, were described in six guidelines [9, 15, 16, 20, 22, 25] either alone [15, 22] or in combination with blood pressure criteria [9, 16, 20, 25]. Of these, altered mental state was cited most frequently [9, 16, 20, 22, 25] with reduced urine output, increased respiratory rate, prolonged capillary refill time, cool peripheries and skin mottling also featuring across multiple guidelines.

Serum lactate was also recommended to identify those at high risk or need for circulatory support using a threshold of > 2 mmol in one study [16] and advocated without specific thresholds in two others [9, 20].

### Fluid type, volume and rate

Preference for crystalloids for initial resuscitation was prevalent [9, 15, 16, 18, 22, 23, 26] and only one early guideline promoted colloid as equally or more effective [20]. In one of the guidelines tailored to management of sepsis in resource-limited settings [22], no specific recommendations were made regarding the relative efficacy of crystalloids or colloids; however, the authors acknowledged that “considering high costs, the risk of allergies and potential renal and coagulopathic side effects of colloids, crystalloid solutions appear more suitable”. Three guidelines suggest consideration of human albumin solution as a second-line fluid choice in those patients with refractory shock or requiring large volumes of crystalloid solutions [9, 16, 23]. Five guidelines specifically recommend administering fluids using a fluid challenge technique during initial resuscitation, using boluses of between 250 and 1000 ml [16, 19, 20, 22, 25].

Specific recommendations on total initial volume of fluid for resuscitation included 30 ml/kg, “at least 20ml/kg” and a note to aggressively treat with estimated 24-h requirement of up to 4 l [15, 22]. Hollenberg et al. suggest 6–10 l in the first day would be typical, titrated by fluid bolus [20]. WHO guidelines were more liberal: 1 l as a bolus and up to 60/ml/kg in the first 2 h [26]. More conservative were NICE who recommended 2× 500 ml boluses rapidly, followed by senior review if no clinical improvement [16]. Two guidelines made no relevant volume recommendations, although titration to fluid challenges was promoted in one [17, 19].

### Assessing response/targets of resuscitation

Two guidelines made no specific recommendations on targets or response assessment [17, 18]. Lactate was promoted for assessment of adequate response in three documents, either alone [19] or in combination with clinical signs [16, 23]. Specific thresholds included a 20% reduction in serum lactate over the first hour [16] and either absolute values of ≤ 1.5 mmol/L or a decrease in non-specified time period [23].

Sequential evaluation of dynamic variables was promoted, including passive leg raise and cardiac ultrasound in ventilated patients [9, 15, 19]. Clinical measures of adequate tissue perfusion (capillary refill, skin temperature and degree of mottling, pulse, blood pressure and conscious level) were advocated in two guidelines specifically tailored to LIC [15, 22]. Earlier guidelines implied additional invasive monitoring (pulmonary artery occlusion pressure, titration to CVP and cardiac output) following a treat-reassess cycle with quarter-hourly boluses of 250–500 ml [20, 23]. SvO_2_ monitoring was advocated in two guidelines [19, 23]. The most recent guidelines noted the lack of evidence of improved outcomes related to CVP and SvO_2_ monitoring [9].

### Criteria for termination

Three guidelines gave no specific indication of stop criteria [16–18]. Others suggested care in continuing therapy but were not specific about objective criteria to guide the decision; for example, **“**Fluid resuscitation should be stopped or interrupted when no improvement of tissue perfusion occurs in response to volume loading.” [22] and “possible repeat volume restitution is guided by the effects” [23]. Three guidelines specifically warned of the dangers of fluid overload or pulmonary oedema, with varying degrees of caution: “… even in the context of fluid-responsive patients, fluid management should be titrated carefully” [19], stressing the need for arterial oxygenation monitoring [20] and explicitly cautioning liberal fluids where there was no or limited access to vasopressors and mechanical ventilation [15].

### Vasopressors

Three studies made no specific recommendations on choice of vasopressor [16, 18, 19]. Norepinephrine was identified as the preferred first-line vasopressor therapy in 5 studies [9, 15, 17, 20, 23]. Two studies recommended dopamine or epinephrine [21, 22].

Starting criteria were not specified in one study [17]. In those guidelines that gave them, thresholds of arterial hypotension were the main indicators on which to base commencement of pressor support [9, 15, 21, 23], to be initiated after initial fluid management. Two other studies gave non-specific indications as “persistent tissue hypoperfusion” [22] and inadequate “arterial pressure and organ perfusion” [20]. Only the WHO guidelines specified a fluid volume trigger for consideration of pressors; 60 ml/kg within the first 2 h [21]. Once commenced, the most common target was arterial pressure of MAP 65 mmHg [9, 15, 23], with one guideline suggesting higher targets in chronic pre-existing hypertension [19]. Two studies specifically recommended administration via a central venous line using a syringe or infusion pump when available [9, 15].

### Clinical applicability

**Fig. 2:**
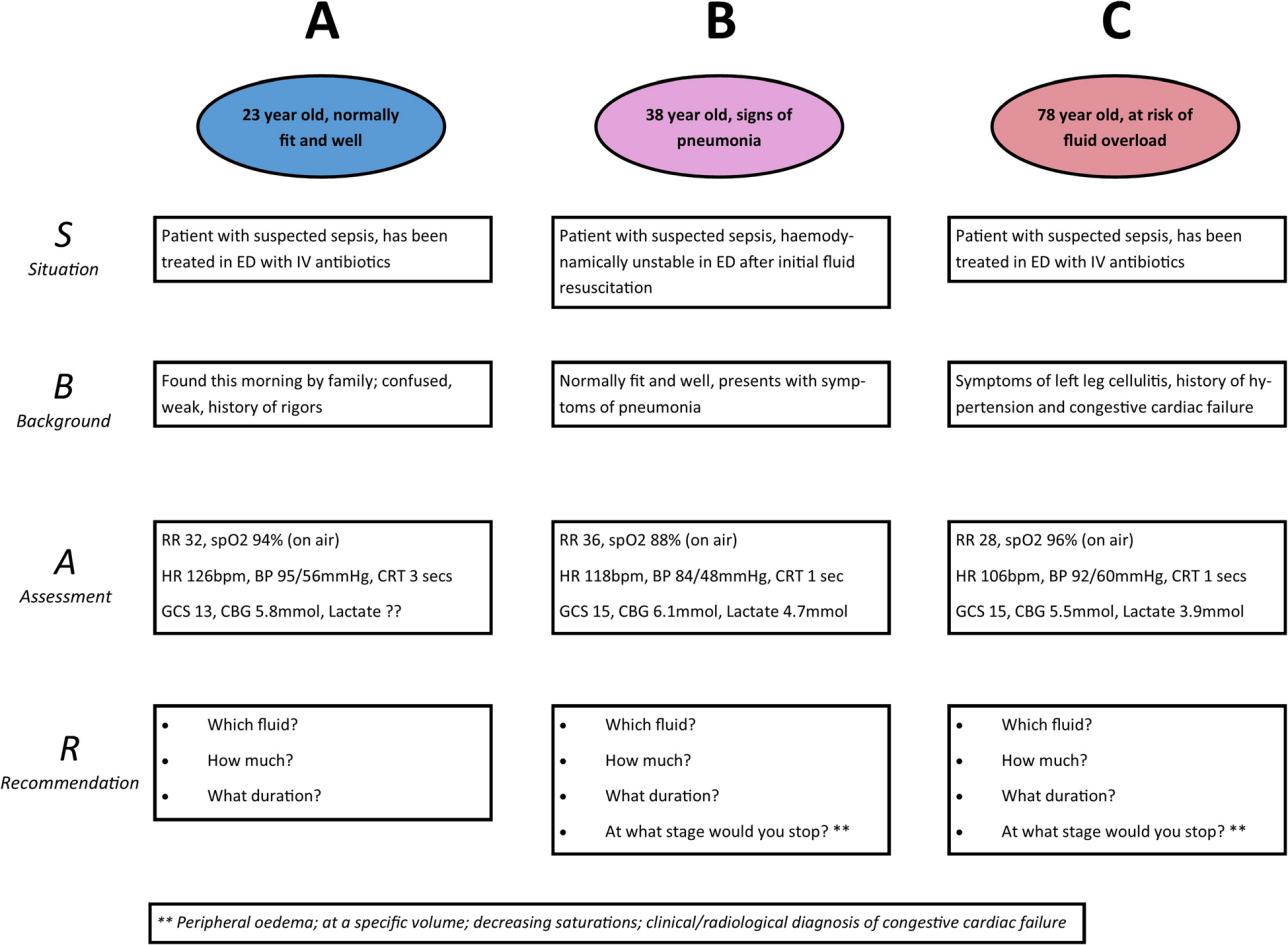
Summary of clinical scenarios

Table 4 describes the applicability of each guideline against the pre-determined assessment scenarios (summarised in Fig. 2, full scenarios in the additional files). Scenario A describes the initial resuscitation of a previously healthy adult patient with suspected infection and evidence of possible hypovolemia *(tachycardia),* poor peripheral perfusion *(cool peripheries, prolonged capillary refill time)* and end-organ dysfunction *(altered mental status)*. In this scenario there is no serum lactate result available.

**Table 4:** Specific fluid therapy recommended in pre-described clinical case scenarios. All guidelines adopt a universal initial approach to fluids (do not take into consideration presenting comorbidities)

Guideline	Scenario A	Scenario B	Scenario C
	*Shock and altered mental status*	*Non-response to initial management, high lactate*	*High lactate and likely congestive cardiac failure*
Cecconi [19]	*Guideline on haemodynamic monitoring in circulatory shock, not specific to sepsis. Recommendations are given in general terms and are not directly applicable to the clinical scenarios.*		
Dunser [22]	> 4 L crystalloid in first 24 h.	No additional specific guidance.	Warning given regarding fluid overload. No fluid if not clinically hypo-perfused.
Hollenberg [20]	250-500 ml boluses over 15 min titrated to clinical endpoints and cardiac measures of fluid responsiveness. No ceiling given (liberal).	No additional specific guidance.	No additional specific guidance.
Misango [15]	30 ml/kg crystalloid over 3 h, continue if fluid responsive.	Peripheral perfusion guided therapy.	Peripheral perfusion guided therapy. Clinical examination to detect overload.
Moller [17]	*Guideline on choice of first-line vasopressor, no specific recommendations relevant to the clinical scenarios.*		
NICE [16]	No definitive guide without lactate.	500 ml crystalloid over < 15 min. Seek senior help at 2 L.	500 ml bolus in response to high lactate, as in scenario B. No specific guidance regarding fluid overload.
Perner [18]	*Guideline on choice of resuscitation fluid, general recommendation for use of crystalloid over other fluid types; no other specific recommendations relevant to the clinical scenarios.*		
Reinhart [23]	500-1000 ml crystalloid over 30 min	Repeat bolus according to response, central monitoring. Target lactate.	Continue and monitor central pressures
Rhodes [9]	30 ml/kg crystalloid over 3 h	Repeat bolus according to response, including invasive and non-invasive monitoring. Target lactate. No volume ceiling given.	Clinical reassessment to detect pulmonary oedema
WHO [25]	1000 mL crystalloid immediately, continued at 20 ml/kg/h (max 60 ml/kg in first 2 h).	Between 2 and 6 h, fluid at 5-10 ml/kg/h if SBP < 90 and signs of poor perfusion continue.	Alert for signs of fluid overload (increased JVP, increasing crackles/rales): reduce rate if present.

Scenario B describes the ongoing resuscitation of a previously healthy adult patient, presenting with symptoms of pneumonia and displaying definite evidence of hypovolemia *(tachycardia, systolic hypotension < 90 mmHg)* and tissue hypoperfusion *(serum lactate > 4 mmol/L)*, demonstrating refractory haemodynamic instability following intravenous administration of 2500 ml of crystalloid.

Scenario C describes the initial resuscitation, as well as criteria for terminating fluid therapy, in an elderly patient with suspected infection and evidence of possible hypovolaemia *(tachycardia, systolic blood pressure < 100 mmHg in the context of known hypertension)*, raised serum lactate and presenting comorbidities suggesting likely fluid intolerance (history of congestive cardiac failure).

Three guidelines gave recommendations that were insufficiently specific to apply to any of the pre-described clinical scenarios [17–19]. Of the remaining seven, all except the NICE guidelines recommended fluid resuscitation with crystalloid in scenario A. The NICE guidelines do not provide a definitive guide to initiating therapy without a lactate measurement [16]. In scenario B, following non-response to initial fluid resuscitation, two guidelines do not provide additional specific guidance, beyond their initial recommendations to resuscitate liberally with crystalloid [20, 22]. Of the five that do make recommendations, three recommend repeat boluses [9, 16, 23], whilst the WHO guideline recommends continuing infusion at 5–10 ml/kg/h [25]. The Misango et al. guidelines for resource-limited settings provide a more general recommendation to continue fluid resuscitation to target clinical surrogate markers of peripheral perfusion [15]. Considered against scenario C, designed to assess recommendations in the management of a patient with likely fluid intolerance, two guidelines do not provide any specific guidance regarding termination of fluid therapy in the context of clinical volume overload [16, 20]. Four of the remaining five guidelines recommend clinical reassessment to detect fluid overload and/or pulmonary oedema [9, 15, 22, 25], with the WHO guideline recommending a reduction in the rate of fluid infusion if clinical signs of overload are present. The Reinhart et al. guideline recommends monitoring of central venous pressure alone to detect volume overload [23].

None of the guidelines suggested any refinement for patients with malnutrition.

## Discussion

We identified ten individual guidelines which might be used by clinicians to guide fluid therapy in sepsis in LIC. The contents of recommendations demonstrate a general shift over time in evidence and practice in a number of ways: (1) crystalloid superiority over colloid; (2) lower initial volumes accompanied by dynamic monitoring of response; (3) shift away from CVP and SvO_2_ monitoring as a proxy target for treatment, towards the dynamic assessment of markers of tissue perfusion and end-organ damage; (4) the use of a breadth of clinical signs and markers of end-organ damage to promote treatment escalation; and (5) robust superiority evidence for norepinephrine as first-line pressor in septic shock.

The performance of guidelines against AGREE II criteria varied widely, with overall average scores ranging from 34.8 to 83.0%. Most guidelines performed well in terms of specifically defining their objectives and target population and providing a clear and unambiguous presentation of their final recommendations. Stakeholder involvement was minimal except within NICE and Surviving Sepsis Campaign guidelines, both of which included lay representatives on their guideline development committees and allowed for stakeholder feedback and comment prior to publication [9, 16]. We also identified a concerning lack of explicit consideration of real-world “applicability”, with few guidelines providing explicit guidance on implementing published recommendations or considering the likely resource implications of doing so. These omissions may be particularly relevant to applying guideline recommendations in LIC, where the front-line impact of sparse resources may be particularly acute.

Recommendations for guidelines were almost universally derived from evidence in high-income countries. With three notable exceptions in which it was explicit that guidelines were aimed at LIC [15, 21, 22], there was a presumption of access to intensive care facilities and the recommendations were not refined for areas in which high-level medical support (ventilation and renal replacement therapy) was not available.

Refinement of treatment protocols at the level of the patient was apparent in the widespread recommendation of an “assess-do-reassess” process for fluids. The measures by which these assessments were ideally made ranged from broadly applicable clinical findings to more recent suggestion of therapy tailored to cardiac output and fluid responsiveness (assessed by ultrasound or another more invasive method). Such methods have been used in middle-income countries as part of the ANDROMEDA study, although limited to regional units [27]. Ultrasound may therefore have a place if training and hardware provision can be met. We note the use of capillary refill and lactate in the same study which might provide accessible ways of refining care at an individual level, with the suggestion that capillary refill would perform at least as well as monitoring of serum lactate in these situations.

We identified some aetiology-specific recommendations, specifically from Misango et al. and the WHO guideline, both of which noted that malaria and dengue represented special circumstances. Caution was noted in guidelines from HIC for those at risk of cardiovascular decompensation with high fluid volumes. It is possible, although untested, that in LIC other patient characteristics might provide ways of refining treatment pathways where facilities are limited. Evidence from treatment of mycobacteraemic sepsis, which is common in sub-Saharan Africa [4] suggests a relative intolerance to fluids. Similarly, HIV is associated with high rates of diastolic heart failure (43% of those on established antiretroviral therapy [28]) and in areas in of high seroprevalence this may become relevant in the majority of admitted patients.

Relatedly, there was a lack of specific recommendations made in the guidelines after the initial fluid bolus and stabilisation period. This is, perhaps, understandable given the highly divergent outcome pathways patients may follow. However, evidence that de-escalation of fluid therapy has benefits in morbidity might be more widely recognised if this issue was addressed in published sepsis guidelines. Beyond the use of fluids, centrally given norepinephrine was almost unanimously agreed to be the best initial vasoactive agent. This gives rise to a clinical deficit in low-income countries in which central venous access is rare and potentially dangerous.

We have adopted strict protocols in this systematic review, including dual extraction and synthesis. Another strength is the use of the validated AGREE II tools to describe the guideline development in multiple domains. Lastly, we have endeavoured to objectively measure the bedside utility by assessing the guidelines against structured clinical scenarios and believe that this novel method represents a further dimension in which to gauge the worth of the guidelines.

We have described guidelines over almost two decades and have summarised data agnostic of the year of publication; it is reasonable to believe that more recent recommendations are better supported by evidence and are therefore superior. This emphasises the need for explicit dates for update or retirement of all guidelines. We are also unable to map which guidelines are currently used and how discrepancies are resolved at the level of the hospital or clinician. Our search did not include local hospital policies and our assumption that these are likely to be related to one of the published guidelines may not be correct.

We have also assumed that codifying patient presentation and the markers by which they are assessed can be done without resort to “physician impression and tailored therapy”. This tension is present in all guidelines, but we feel there is considerable evidence that systematising care is beneficial for patient outcomes. Improving the specificity of assessment, perhaps using decision support aids, could help clinicians and health service managers in remote and underserved areas.

## Conclusions

Guideline development is a major undertaking. We have noted the robust methodology, including systematic reviews incorporated into the 2 largest and best funded programmes: the NICE and Surviving Sepsis Campaign guidelines.

Given the burden of sepsis in sub-Saharan Africa and across LIC, together with the significant heterogeneity in clinical practice and the emergence of data which suggest we should re-evaluate guidelines in contexts without intensive care, we feel further high-quality, evidence-based and implementable recommendations for fluid management strategies in patient with sepsis in resource-limited settings are urgently required. This could be incorporated into ongoing international efforts to make sepsis guidelines truly global.

## Supplementary information

**Additional file 1.** Exclusion of studies by criterion and emerging issues identified on review of full manuscripts leading to exclusion.

**Additional file 2.** Details of consensus scoring in AGREE-II assessment, where divergence existed between initial scores assigned by individual reviewers.

**Additional file 3.** Detailed overview of manuscripts’ recommendations for fluid administration in sepsis.

**Additional file 4.** Unabridged clinical scenarios 1, 2 & 3, as summarised in Figure 2 in the main manuscript.

## Data Availability

Data sharing is not applicable to this article as no datasets were generated or analysed during the current study.

## Abbreviations

BP: Blood pressure
CVP: Central venous pressure
HIC: High-income countries
HIV: Human immunodeficiency virus
LIC: Low-income countries
MAP: Mean arterial pressure
NICE: National Institute for Health and Care Excellence (UK)
SSA: Sub-Saharan Africa
SSC: Surviving Sepsis Campaign
SvO_2_: Mixed venous oxygen saturation
WHO: World Health Organization
