# Supplementary material for: Recommendations for fluid management of adults with sepsis in sub-Saharan Africa: a systematic review of guidelines
Source: Crit Care. 2020 Jun 5;24:286. doi: 10.1186/s13054-020-02978-4 (PMC7275525; doi:10.1186/s13054-020-02978-4)
Supplement: Supplementary file 1 — Additional file 1. Exclusion of studies by criterion and emerging issues identified on review of full manuscripts leading to exclusion. [file 13054_2020_2978_MOESM1_ESM.docx]

**Exclusion of studies by criterion**

Guidelines, full text not available in English

**(n=6)**

Guidelines, applying to specific populations other than those considered by this review and/or applying to narrow range of clinical scenarios

**(n=7)**

Guidelines, superseded by a more recent edition

**(n=20)**

Summaries or reviews of existing guidelines

**(n=73)**

Expert viewpoint articles by single author or group of authors

**(n=23)**

Observational studies or case series

**(n=93)**

Systematic reviews and/or meta-analyses rather than published guidelines or recommendations for practice

**(n=264)**

**Excluded studies**

Studies included in longlist following consensus discussion between reviewers and inclusion of studies identified outwith database search (as described in Methodology)

**(n=499)**

**Studies included in final review and assessed for quality**

**(n=10)**

**Emerging issues identified leading to study exclusion**

| **Study excluded** | **Rationale** |
| --- | --- |
| Aitken et al. (2011) *Nursing considerations to complement the Surviving Sepsis Campaign guidelines* | Derivative from Surviving Sepsis Campaign guidelines, which are included in final review. Also aimed at nursing rather than clinical care of patients. |
| Pottecher et al. (2006) *Haemodynamic management of severe sepsis: recommendations of the French Intensive Care Societies (SFAR/SRLF) Consensus Conference, 13 October 2005, Paris, France* | On closer examination, full translation of manuscript into English not available. |
| Singer et al. (2016) *The Third International Consensus Definitions for Sepsis and Septic Shock (Sepsis-3)* | A definition of sepsis according to international consensus, rather than a guideline for management. |
| Spapen et al. (1999) *Consensus statement of the treatment of septic shock* | Despite originally including all studies published after 1990, on full review of this manuscript recommendations were found to be so far removed from current clinical practice as to not be of relevance when answering the review question. |
