# Supplementary material for: Recommendations for fluid management of adults with sepsis in sub-Saharan Africa: a systematic review of guidelines
Source: Crit Care. 2020 Jun 5;24:286. doi: 10.1186/s13054-020-02978-4 (PMC7275525; doi:10.1186/s13054-020-02978-4)
Supplement: Supplementary file 2 — Additional file 2. Details of consensus scoring in AGREE-II assessment, where divergence existed between initial scores assigned by individual reviewers. [file 13054_2020_2978_MOESM2_ESM.docx]

# AGREE-II assessment – consensus scores for individual items

## Only included for those items with initial disagreement of >=3 points*

| **Study** | **Item** | **Initial score – reviewer 1** | **Initial score – reviewer 2** | **Consensus score** | **Comments** |
| --- | --- | --- | --- | --- | --- |
| *Cecconi et al* | 7 (Evidence search) | 4 | 1 | 2 | No named database, no inclusion of search terms. Search strategy not provided. Unclear who performed literature search. |
|  | 10 (Formulating recommendations) | 6 | 2 | 5 | Panel of 12 experts employed modified Delphi approach. No indication how consensus was reached, evidence of process or outcome of any voting procedure. |
|  | 23 (Competing interests) | 7 | 4 | 5 | Conflicts of interest recorded as “none”. No indication how this information was sought, or discussion of types of CoI considered. |
| *Dunser et al* | 6 (Target users) | 7 | 4 | 6 | Specifies setting in which recommendations are to apply and how they fit in with other guidelines. Describes target users as “clinicians” but gives no more detail. |
|  | 19 (Implementation tools) | 6 | 3 | 5 | Specific implementation section in guideline. Care bundles presented in table. Flowchart/summary of recommendations in manuscript. Various implementation strategies presented, vague on detail/specifics. |
|  | 20 (Resource implications) | 7 | 3 | 3 | Details of cost consideration in formulation of recommendations theoretical rather than specific cost analyses. No real consideration of resource implications of application of recommendations. |
|  | 22 (Funding source) | 7 | 1 | 6 | Statement that “no external funding” used. Unclear what constitutes ‘external’. Was authors’ time donated or funded. If funded, how? |
|  | 23 (Competing interests) | 7 | 4 | 5 | Statement that “no author has a conflict of interest”. No indication how this information was sought, or discussion of types of CoI considered. |
| *Hollenberg et al* | 10 (Formulating recommendations) | 5 | 2 | 5 | Experts reviewed literature, classified QoE, recomms formulated and debated. Task force chairman modified until <10% disagreement. Who formulated recomms? Voting process to achieve consensus? Results? Mods? |
|  | 11 (Benefits vs risks) | 7 | 3 | 6 | Complications section for each group of interventions considering risks/potential adverse effects. |
|  | 19 (Implementation tools) | 4 | 1 | 3 | No implementation section or other advice on strategies to implement recommendations. Algorithm/flowchart included in manuscript summarising recommendations. |
| *Misango et al* | 13 (External review) | 4 | 1 | 2 | “External peer review was provided through the complete panel of the Global Intensive Care Working Group”. All authors are members of the Group. Not really external. |
|  | 20 (Resource implications) | 7 | 1 | 5 | No details of methods of identification of cost information, or systematic consideration of this in formulation of recommendations. However, resource implications of implementing guidelines acknowledged and alternative options given based on resources available. |
| *Møller et al* | 10 (Formulating recommendations) | 5 | 1 | 4 | Describes general factors considered in formulation of recommendations. “The group agreed on all recommendations”. No specific description of development process or how consensus achieved. |
|  | 15 (Specific recommendations) | 6 | 1 | 6 | Clear statement of recommended action. Potential benefits detailed. Description of caveats where recommendations would not apply. No specific dosage/administration information given. |
| *NICE* | 5 (Views of target population) | 5 | 1 | 3 | Members of public incorporated into Guideline Development Group as lay members (x2). Their role is not described, unclear how their views were sought. No description of what information was gathered or if/how it influenced recommendations. |
|  | 14 (Updating procedure) | 6 | 3 | 6 | Guideline developed according to *NICE guideline development manual* (separate document). This manual specifies process for updating guidelines; development team will conduct literature search to identify any new evidence and update if necessary. Updates prioritised according to users’ need. No specific time interval given. |
|  | 21 (Audit criteria) | 2 | 6 | 2 | Template provided for baseline assessment of practice. Generic advice and resources provided on monitoring implementation of NICE guidance. No specific auditing standards defined, nor guidance on frequency/interval of measurements. |
| *Perner et al* | 6 (Target users) | 3 | 6 | 4 | Strong implication from detailed description of target population, setting, clinical context. However target audience of guideline never specifically articulated. |
| *Rhodes et al* | 14 (Updating procedure) | 1 | 4 | 1 | This guideline is, in itself, an update of a previous iteration of SSC. However there is no specific statement in the manuscript that this guideline will be updated nor is any procedure described. |
|  | 21 (Audit criteria) | 3 | 7 | 5 | Supplementary document produced by Surviving Sepsis Campaign *(Implementation Guide)* provides guidance on process of quality improvement, groups interventions into ‘bundles’ with suggested targets (i.e. standards of care). No specific reference to this document in the manuscript. Targets defined at a patient level, however nothing specified at institutional level (e.g. % of patients meeting patient-level targets). |
