# Supplementary material for: Recommendations for fluid management of adults with sepsis in sub-Saharan Africa: a systematic review of guidelines
Source: Crit Care. 2020 Jun 5;24:286. doi: 10.1186/s13054-020-02978-4 (PMC7275525; doi:10.1186/s13054-020-02978-4)
Supplement: Supplementary file 3 — Additional file 3. Detailed overview of manuscripts’ recommendations for fluid administration in sepsis. [file 13054_2020_2978_MOESM3_ESM.docx]

**Overview of guidelines decision to prescribe fluids for sepsis**

| **Author** | **Summary** | **Sepsis definitions if provided (explicit or implied)** | **Fluid recommendations** | **Fluids indicated in clinical scenario (based on criterion)** | | |
| --- | --- | --- | --- | --- | --- | --- |
| Cecconi  (2014)[1] | ESICM panel guidelines on circulatory shock. Addressed 5 specific questions related to haemodynamic monitoring. Not restricted to septic shock. | Abstract definition, no physiological parameters stated^[[1]](#footnote-1)^ | Not specified |  |  |  |
| Moller  (2016)[23] | Scandinavian guidelines on vasopressor use in acute circulatory failure | Not explicit | None relevant |  |  |  |
| Perner  (2014)[24] | Scandinavian guidelines on choice of fluid for resuscitation in acute circulatory failure | Not explicit | General: crystalloids recommended |  |  |  |
| Dunser  (2012)[4] | Recommendations for sepsis management in resource limited settings | Sepsis-2 (modified to remove WCC, replacing with “malaise and/or apathy”)^[[2]](#footnote-2)^ | Fluids indicated if: tissue hypoperfusion.^[[3]](#footnote-3)^  Target SBP >90 and good peripheral perfusion. | Yes (CRT) | Yes (SBP) | No (lactate not used) |
| Hollenberg  (2004)[5] | American practice guidelines for haemodynamic support of adults with sepsis | Haemodynamic support considered for hypoperfusion (SBP<90, MAP<65, fall SBP>40, change in mental status, decrease in urine output, increased lactate. |  | Yes (confusion) | Yes (SBP, MAP) | Yes (lactate) |
| Misango  (2017)[22] | Recommendations for haemodynamic assessment and support in sepsis and septic shock in resource-limited settings | Not explicit | If hypovolaemic / fluid responsiveness defined by passive leg raise^[[4]](#footnote-4)^  Target: tissue perfusion (CRT, skin temperature) | Yes (CRT) | No (lack of peripheral perfusion data) | No (lack of peripheral perfusion data) |
| NICE  (2016)[7] | UK practice guidelines on sepsis recognition, diagnosis and early management | Clinical suspicion of infection, with risk criteria for death (e.g. altered mental status, evidence of microvascular perfusion defect [mottled/delayed CRT]; high respiratory rate) | If SBP <90 or any high risk criteria and lactate >4. | No (lactate unknown) | Yes (SBP, lactate) | No (no high risk features) |
| Reinhart  (2010)[9] | Clinical practice guidelines of the German Sepsis Society | Sepsis-2 | Target: “haemodynamic stabilization”.  Otherwise not specified. |  |  |  |
| Rhodes  (2016)[13] | Surviving Sepsis Campaign - International guidelines for the management of sepsis and septic shock | Sepsis-3^[[5]](#footnote-5)^ | Target: stabilise sepsis-induced hypoperfusion / organ dysfunction. | Yes (SBP, SOFA) | Yes (SOFA, lactate) | Yes (lactate) |
| WHO  (2011)[25] | WHO guidelines for the management of common illnesses in resource limited settings | Severe sepsis / septic shock: suspected infection plus hypotension (SBP <90) plus ≥1 of: pulse >100; resp rate >24; temperature <36^o^C or >38^o^C. |  | No (“not shocked”) | Yes (SBP) | No (“not shocked”) |

#

1. “Life-threatening, generalized form of acute circulatory failure associated with inadequate oxygen utilization by the cells” [↑](#footnote-ref-1)
2. Sepsis-2: Proven or highly suspected infection plus presence of ≥2 of the following conditions: heart rate >90 bpm; respiratory rate or PaCO_2_ <32 mmHg; temperature <36 or>38^o^C; White blood cell count <4×10^6^ or >12×10^6^ g/L or >10% immature forms. Severe sepsis = sepsis plus; additional confusion, hypoxaemia, elevated lactate.

   SBP = systolic blood pressure (mmHg); MAP = mean arterial blood pressure (mmHg) [↑](#footnote-ref-2)
3. Tissue hypoperfusion indicated by: prolonged capillary refill; skin mottling; cool peripheries; weak peripheral pulses; altered mental status; UO <0.5ml/kg/hr. [↑](#footnote-ref-3)
4. Passive leg raise test indicates fluid responsiveness if change in pulse pressure of >15% [↑](#footnote-ref-4)
5. qSOFA ≥2 [↑](#footnote-ref-5)
