# Supplementary material for: Recommendations for fluid management of adults with sepsis in sub-Saharan Africa: a systematic review of guidelines
Source: Crit Care. 2020 Jun 5;24:286. doi: 10.1186/s13054-020-02978-4 (PMC7275525; doi:10.1186/s13054-020-02978-4)
Supplement: Supplementary file 4 — Additional file 4. Unabridged clinical scenarios 1, 2 & 3, as summarised in Figure 2 in the main manuscript. [file 13054_2020_2978_MOESM4_ESM.docx]

# Clinical Scenarios

## Sepsis Scenario 1 – Initial therapy in a fit person

### Setting

Emergency department

### History

- 23-year-old found this morning by family in weak state, following a week history of fever and rigors. Estimated body mass 60kg
- PMH: Nil
- Drugs: Nil
- Allergies: Nil known

### Presentation

- A - patent
- B - RR 32, Sp02 94% on air, equal air entry - clear
- C - HR 126, BP 95/56, cool peripheries - CRT 3 sec, temp 35.2C, lactate unavailable
- D - GCS 13 (E3, V4, M5), BM 5.8
- E – nil

### Clinical progress

- In resus
- Has been given ceftriaxone intravenously

### Fluid question

- Which fluid you would give?
- How much?
- Over what duration?

### Clinical progress 2

- A - patent
- B - unchanged
- C - unchanged
- D - unchanged
- E - unchanged

### Fluid question

- Which fluid you would give?
- How much?
- Over what duration?

Repeat Clinical non-progress

Repeat scenario with low weight (40kg)

## Sepsis Scenario 2 – Non-response at 2l in a fit person

### Setting

Emergency department

### History

- 38-year-old with symptoms of pneumonia, estimated body mass 100kg
- PMH: Nil
- Drugs: Nil
- Allergies: Nil known

### Presentation

- A - patent
- B - RR 36, Sp02 88% on air, left basal bronchial breath sounds
- C - HR 118, BP 84/48, warm peripheries - CRT 1 sec, temp 39.2C, lactate 4.7mmol/l
- D - GCS 15, BM 6.1
- E – nil
- No ITU / other help available

### Clinical progress

- In resus, oxygen therapy started targeting SpO_2_ to 94-98%
- Has been given antibiotics intravenously (following hospital policy)
- Has been given, over the first 2 hours of resuscitation, 2000ml crystalloid

### Fluid question

- Which fluid you would give?
- How much?
- Over what duration?

### Clinical progress 2

- A - patent
- B - unchanged
- C - unchanged
- D - unchanged
- E – unchanged
- No ITU / other help available

### Fluid question

- Which fluid you would give?
- How much?
- Over what duration?
- At what stage would you stop? – which of the following
  - Peripheral oedema
  - At a specific volume
  - Decreasing saturations
  - Clinical diagnosis of CCF
  - Radiological diagnosis of CCF

## Sepsis Scenario 3 – A patient at risk of CCF

### Setting

Emergency department

### History

- 78-year-old with symptoms of cellulitis, estimated body mass 60kg
- PMH: Hypertension, congestive cardiac failure, well controlled
- Drugs: Unknown
- Allergies: Nil known

### Presentation

- A - patent
- B - RR 28, Sp02 96% on air, chest clear
- C - HR 106, BP 92/60, warm peripheries - CRT 1 sec, temp 38.6C, lactate 3.9mmol/l
- D - GCS 15, BM 5.5
- E – cellulitis left leg below knee

### Clinical progress

- Has been given benzylpenicillin and flucloxacillin intravenously (following hospital policy)
- No fluid given yet

### Fluid question

- Which fluid you would give?
- How much?
- Over what duration?

### Clinical progress 2

- A - patent
- B - unchanged
- C - unchanged
- D - unchanged
- E – unchanged
- No ITU / other help available

### Fluid question

- Which fluid you would give?
- How much?
- Over what duration?
- At what stage would you stop? – which of the following
  - Peripheral oedema
  - At a specific volume
  - Decreasing saturations
  - Clinical diagnosis of CCF
  - Radiological diagnosis of CCF
